# The role of SIRT3 in homeostasis and cellular health

**DOI:** 10.3389/fncel.2024.1434459

**Published:** 2024-08-02

**Authors:** Dennison Trinh, Lina Al Halabi, Harsimar Brar, Marie Kametani, Joanne E. Nash

**Affiliations:** ^1^Department of Biological Sciences, University of Toronto, Toronto, ON, Canada; ^2^Department of Biological Sciences, University of Toronto Scarborough Graduate Department of Cells Systems Biology, University of Toronto Cross-Appointment with Department of Psychology, University of Toronto Scarborough Scientist – KITE, Toronto, ON, Canada

**Keywords:** SIRT3, mitochondria, homeostasis, allostasis, proteostasis, metabolism, immune response, oxidative stress

## Abstract

Mitochondria are responsible for maintaining cellular energy levels, and play a major role in regulating homeostasis, which ensures physiological function from the molecular to whole animal. Sirtuin 3 (SIRT3) is the major protein deacetylase of mitochondria. SIRT3 serves as a nutrient sensor; under conditions of mild metabolic stress, SIRT3 activity is increased. Within the mitochondria, SIRT3 regulates every complex of the electron transport chain, the tricarboxylic acid (TCA) and urea cycles, as well as the mitochondria membrane potential, and other free radical scavengers. This article reviews the role of SIRT3 in regulating homeostasis, and thus physiological function. We discuss the role of SIRT3 in regulating reactive oxygen species (ROS), ATP, immunological function and mitochondria dynamics.

## Introduction

The Sirtuins (SIRTs) are a family of NAD + -dependent deacetylases and ribosyltransferases (Michan and Sinclair, 2007; Huang et al., 2010; Li and Kazgan, 2011) linked with cytoprotective function and extended lifespan (Kaeberlein et al., 1999; Howitz et al., 2003; Law et al., 2009; Benigni et al., 2019). The beneficial effects of SIRTs have been observed in yeast, experimental invertebrates and vertebrates, as well as humans (Kaeberlein et al., 1999; Albani et al., 2014). In mammals, there are seven SIRT (SIRT1-7) family members, each with unique subcellular distribution and function (Michan and Sinclair, 2007; Jesko et al., 2017). SIRT1, SIRT6, and SIRT7 are localised to the nucleus, with roles in cell metabolism/inflammation, DNA repair/metabolism, and rRNA transcription, respectively (Mostoslavsky et al., 2006; Grob et al., 2009; Herranz et al., 2010; Herskovits and Guarente, 2013; Garcia-Rodriguez et al., 2014; Ryu et al., 2014; Wang X. X. et al., 2016; Santos-Barriopedro and Vaquero, 2018). SIRT2, which localises to both the nucleus and cytoplasm, is involved in the regulation of the cell cycle (Skoge et al., 2014; Xu D. et al., 2019). Lastly, SIRT3, SIRT4, and SIRT5 are localised to the mitochondria where they have functions in processes such as metabolism, insulin secretion, and ammonia detoxification respectively (Haigis et al., 2006; Ahn et al., 2008; Du et al., 2011; Herskovits and Guarente, 2013; Zhu et al., 2014; Li J. et al., 2016).

In mammals, the SIRT family evolved from the yeast homolog Sir2. The Silent Information Regulator (Sir) proteins, comprising Sir1-4, are essential in the transcriptional repression of *HML and HMR*, which are silent mating type loci, important for governing cell-type identity and the sexual cycle (Sharp et al., 2003). In yeast, Sir2 has been linked to longevity, where mutations affecting the function of Sir2 result in reduced life span and inability to reduce detrimental recombination in rDNA (Tissenbaum and Guarente, 2001; Rogina and Helfand, 2004; Burnett et al., 2011). Conversely, increasing Sir2 gene expression in WT yeast cells extends life span (Anderson et al., 2003). Through the regulation of these processes, SIRTs have been linked to longevity in organisms such as *C. elegans* to humans (Anderson et al., 2003; Rose et al., 2003; Benigni et al., 2019). While several Sirtuins have been linked with beneficial effects, the focus of this review is SIRT3.

**Figure 1:**
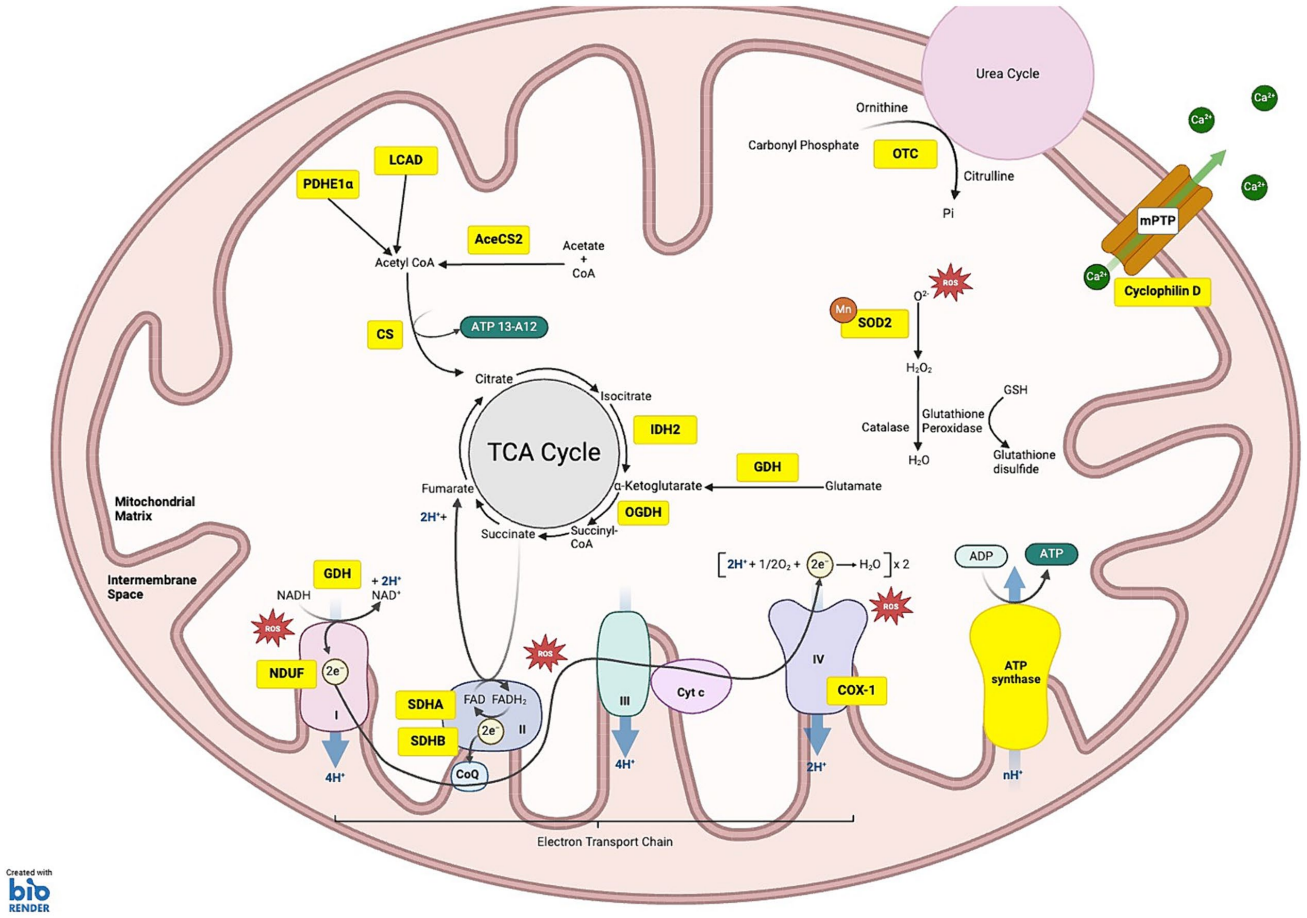
SIRT3 substrates under homeostatic conditions: the schematic illustrates the various substrates and pathways regulated by SIRT3. **(1) OXIDATIVE PHOSPHORYLATION:** SIRT3 targets substrates across all five complexes of the electron transport chain (ETC), regulating their function and efficiency. Within complex I NDUF (NADH oxidoreductase), SIRT3 deacetylates NDUFA11 and NDUFS8 subunits. Complex II: succinate dehydrogenase (SDH), SIRT3 deacetylates the SDHA and SDHB subunits, regulating ATP production from succinate oxidation. Complex III, SIRT3 is critical for balancing ATP homeostasis and oxidative stress regulation, although specific subunits affected by deacetylation are not fully characterized. Complex IV, SIRT3-mediated deacetylation of COX-1 enhances mitochondrial stability and reduces apoptosis. Complex V: ATP synthase, SIRT3 deacetylation improves the efficiency of ATP synthesis. **(2) METABOLISM OF PROTEINS, CARBOHYDRATES AND PROTEINS**: deacetylation of long-chain acyl-CoA dehydrogenase (LCAD) and Lower case pyruvate dehydrogenase E1α (PDHE1 α) aid fatty acid oxidation and ketogenesis. SIRT3 deacetylates acetyl-CoA synthetase 2 (ACS2), which is involved in acetyl-CoA production. Citrate synthase (CS) is activated by SIRT3, promoting the condensation of acetyl-CoA with oxaloacetate to form citrate. SIRT3 deacetylates isocitrate dehydrogenase 2 (IDH2) causing conversion of isocitrate to α-ketoglutarate, a crucial step that helps maintain the flow of the TCA cycle. Additionally, 2-oxoglutarate dehydrogenase (OGDH) is deacetylated by SIRT3, increasing its ability to convert α-ketoglutarate into succinyl-CoA. **(3) ROS DETOXIFICATION:** SIRT3 deacetylates manganese superoxide dismutase (MnSOD). **(4) MITOCHONDRIAL MEMBRANE:** SIRT3-induced deacetylation of Cyclophilin D reduces pore opening, preventing depolarization of the mitochondrial membrane. **(5) UREA CYCLE:** SIRT3 deacetylates ornithine transcarbamylase (OTC), converting carbamoyl phosphate and ornithine into citrulline. **KEY** Yellow boxes: Enzymes and substrates directly regulated by SIRT3; Red stars: Sites of reactive oxygen species (ROS) production.

Within mitochondria, SIRT4 and SIRT5 are ADP ribosylases and SIRT3 is the major protein deacetylase. In SIRT3 KO mice, liver extracts reveal a 63% increase in mitochondrial protein acetylation, which is not observed in SIRT4 or SIRT5 KO mice (Lombard et al., 2007). This hyperacetylation of mitochondrial proteins has also been observed in the kidneys and muscles of SIRT3 KO mice (Fernandez-Marcos et al., 2012). In mitochondria, SIRT3 is a nutrient sensor, serving to regulate and stabilize mitochondria, and thus maintain homeostasis of the entire cell. SIRT3 is activated by cellular stress, stabilizing energy production (e.g., ATP and NAD), and also maintaining homeostasis through regulation of oxidative stress, mitochondrial dynamics, and proteostasis (Gleave et al., 2017; Tyagi et al., 2018; Li et al., 2019; Lee et al., 2021; Zhang et al., 2021). SIRT3 has hundreds of substrates involved in the regulation of gene expression, energy homeostasis, and oxidative stress (Figure 1) (Hallows et al., 2008; Kincaid and Bossy-Wetzel, 2013; Vassilopoulos et al., 2014; Liu et al., 2015; Cheng et al., 2016; Novgorodov et al., 2016; Yu et al., 2017). The broad-spanning effects of SIRT3 in both the healthy and diseased state are the focus of this review.

SIRT3 is transcribed as a 44 kDa inactive protein in the nucleus, then targeted to the mitochondria by a mitochondrial targeting sequence (MTS). Within the mitochondria, the N-terminus of SIRT3 is cleaved, producing a 28 kDa active protein (Schwer et al., 2002). Certain mutations in SIRT3 have been linked with altered physiological function. For example, in male populations, the single nucleotide polymorphism (SNP) G477T in SIRT3 is linked with increased survival (Rose et al., 2003). Furthermore, variable number of tandem repeats (VNTR) polymorphisms, which enhance SIRT3 expression are almost always present in individuals over the age of 90 (Bellizzi et al., 2005).

**Figure 2:**
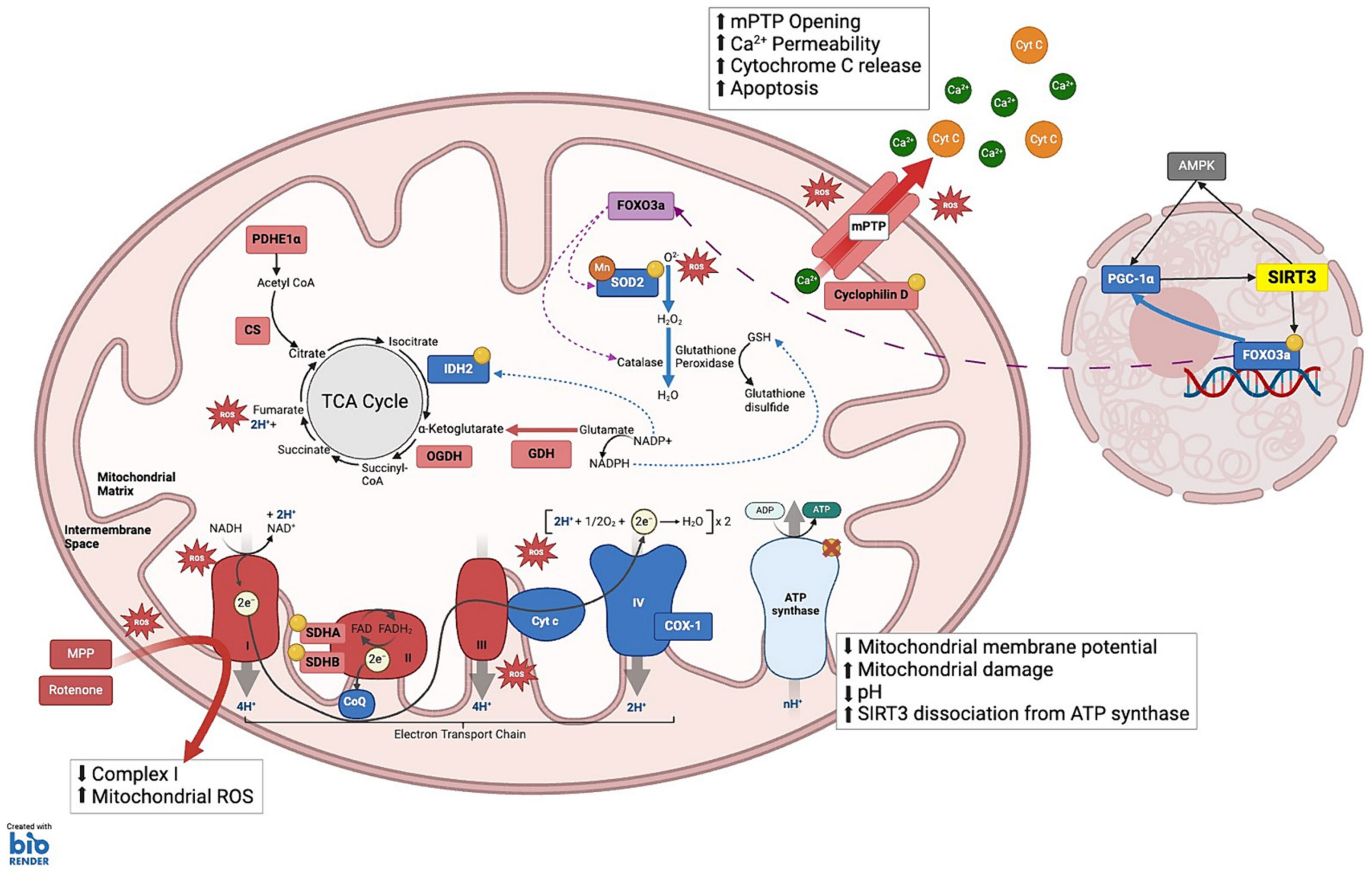
SIRT3 substrates under cellular stress: This schematic illustrates the complex interplay between various mitochondrial components and SIRT3 to maintain cellular energy homeostasis and mitigate oxidative stress. **(1) TCA CYCLE**: Isocitrate dehydrogenase 2 (IDH2) catalyzes the oxidative decarboxylation of isocitrate to α-ketoglutarate; Manganese superoxide dismutase (MnSOD), enabling the conversion of ROS into hydrogen peroxide (H₂O₂); Glutamate dehydrogenase (GDH) enhances the conversion of glutamate to α-ketoglutarate and ammonia, while simultaneously reducing NADP+ to NADPH. Elevated NADPH levels facilitate the reduction of oxidized glutathione to reduced glutathione (GSH). Pyruvate dehydrogenase E1α (PDHE1α) and GDH: SIRT3 modulates the activity of these enzymes through allosteric inhibition by metabolic products: NADH inhibits citrate synthase (CS), isocitrate dehydrogenase (IDH), and 2-oxoglutarate dehydrogenase (OGDH); ATP inhibits IDH; and succinyl-CoA inhibits OGDH and CS. **(2) ELECTRON TRANSPORT CHAIN** Complex I: NDUFA11 and NDUFS8; Complex II: SDHA and SDHB, enhancing the transfer of electrons from succinate to ubiquinone (CoQ), thus linking the TCA cycle to the ETC; Complex IV: Cytochrome c Oxidase 1 (COX-1) maintains electron transfer efficiency and prevents oxidative stress-induced apoptosis; Complex V: ATP synthase, which is crucial during periods of high energy demand, particularly in tissues such as neurons and muscles. **(3) MITOCHONDRIA MEMBRANE INTEGRITY AND APOPTOSIS PREVENTION:** SIRT3 maintains mitochondrial integrity by deacetylating Cyclophilin D, a key regulator of the mitochondrial permeability transition pore (mPTP). This action prevents the opening of mPTP, thereby averting the collapse of the mitochondrial membrane potential and inhibiting the release of pro-apoptotic Cytochrome c (Cyt C) into the cytosol. **(4) MITOCHONDRIA TRAFFICKING AND STRESS RESPONSE:** SIRT3 influences the translocation of deacetylated Forkhead box O3a (FOXO3a) to the mitochondria, where it activates antioxidative enzymes such as Catalase and MnSOD, reducing ROS and mitigating oxidative stress. **KEY** Yellow circles: Enzymes and substrates directly regulated by SIRT3; Green: Sites of ROS clearance; Red: Sites of reactive oxygen species (ROS) production; Green dashed-arrows: action of NADP+/NADPH that aid ROS clearance; Purple dashed-arrows: Trafficking from nucleus to the mitochondria to activate antioxidant enzymes.

SIRT3 activity is elevated during conditions of cellular stress, upregulating processes such as energy production and ROS scavenging to help maintain cellular homeostasis (Figure 2) (Chen et al., 2011; Li et al., 2015; Zhang et al., 2016). However, SIRT3 levels, and thus activity, declines with age, and is further decreased in pathologies such as cardiac myopathy, fatty liver disease, and neurodegenerative diseases, such as Alzheimer’s, Huntington’s and Parkinson’s disease (Kendrick et al., 2011; Fu et al., 2012; Weir et al., 2012; Yang et al., 2015; Kwon et al., 2017; Yu et al., 2017; Song et al., 2020, 2021; Trinh et al., 2023). Due to the crucial role that SIRT3 plays in adapting to metabolic stress, reduced SIRT3 activity has also been linked with metabolic syndrome. Metabolic syndrome increases the risk of diabetes through insulin resistance, obesity, hypertension Alzheimer’s and cardiovascular diseases (Pugazhenthi et al., 2017; Tyagi et al., 2022). Together, these studies suggest that elevated levels of SIRT3 are protective and linked with increased longevity, whereas a decline in SIRT3 levels are linked with cellular stress and aging and disease (Choudhury et al., 2011; Kendrick et al., 2011; Fernandez-Marcos et al., 2012; Chen et al., 2013; Li L. et al., 2016; Yang Y. et al., 2016; Wei et al., 2017; Yu et al., 2017; Benigni et al., 2019; Zhang et al., 2020; Song et al., 2021). How SIRT3 mediates beneficial effects in various organ systems will be discussed in this review.

## The role of SIRT3 in homeostasis

### SIRT3 maintains metabolic and energetic stability

Substrates for SIRT3 reside in all metabolic pathways of the mitochondria, such as the tricarboxylic acid (TCA) cycle, fatty acid oxidation (FAO) and the electron transport chain (ETC) (Figure 1). Thus, the metabolic state of the cell governs SIRT3 activity, and is the reason why cellular stress has such a large impact on SIRT3 levels and function. Increased NAD+/NADH levels in the mitochondria, which can result from a decrease in ATP production through the ETC, serves to activate SIRT3 activity and expression. This can occur due to the accumulation of ROS, where mitochondrial uncoupling protein 2 (UCP2) is activated to decrease ROS generation by the ETC, and in turn ATP production as well. Activation of cAMP due to limited ATP also increases NAD+ availability in the mitochondria, further leading to increased SIRT3 activity (Palmeira et al., 2019). Limited glucose also leads to the accumulation of forkhead box O3a (FOXO3a) in the mitochondria as a result of phosphorylation by adenosine monophosphate-activated protein kinase (AMPK), forming a FOXO3a/SIRT3/mtRNAPol complex to upregulate transcription of the ETC complexes (Peserico et al., 2013). The TCA cycle generates energy from the catabolism of glucose and dietary fatty acids. A study comparing SIRT3 KO mice and wild type controls using [1,6-^13^C] glucose administration and *ex vivo* ^13^C-NMR spectroscopy demonstrated the role of SIRT3 in glucose metabolism (Kristian et al., 2021). In this study, SIRT3 KO results in impaired glucose metabolism during the TCA cycle. Furthermore, in the brain of SIRT3 KO mice, generation of downstream glucose metabolites including glutamine, creatine and GABA, are reduced compared to WT in mice (Kristian et al., 2021). While the TCA cycle is crucial for providing substrates for other processes such as the ETC, strict regulation of its activity by metabolic products is essential for preventing unnecessary nutrient metabolism and ROS accumulation (Arnold and Finley, 2023). This is achieved through allosteric inhibition of rate-controlled enzymes citrate synthase (CS), isocitrate dehydrogenase (IDH), and 2-oxoglutarate dehydrogenase (OGDH), whereby high NADH can bind to all three enzymes, high ATP can bind to IDH, and succinyl-CoA binds to OGDH and CS which are regulated by SIRT3 activity (LaNoue et al., 1972; Gabriel et al., 1986; Liu et al., 2018). During the TCA cycle, Acetyl-CoA synthetase 2 (AceCS2) catalyzes the ligation of acetate and CoA to produce acetyl co-enzyme A (acetyl-CoA), which is an important pathway for the aerobic production of ATP as well as intermediate molecules involved in the synthesis of amino and fatty acids, essential for protein and membrane formation (Schwer et al., 2006; Hirschey et al., 2011). SIRT3 reversibly deacetylates the mitochondrial form of AceCS2 at its active site Lys-642, which upregulates AceCS2 activity both *in vitro* and *in vivo* (Schwer et al., 2006). Based on these observations, the TCA cycle can be said to be upregulated in response to low energy availability. Given that aging results in the decrease of adenosine triphosphate (ATP) production (Schapira and Cooper, 1992), SIRT3 plays a critical role in providing energy by facilitating the metabolic use of acetate (Hallows et al., 2006; Schwer et al., 2006). Also, SIRT3-induced acetylation is critical for regulating the activity of other enzymes of the TCA cycle as well, such as pyruvate dehydrogenase E1α (PDHE1α), succinate dehydrogenase (SDH), long-chain acyl-CoA (LCAD), and glutamate dehydrogenase (GDH) (Finley et al., 2011; Santos et al., 2021; Zhang et al., 2021; Fu et al., 2022).

The ETC, located in the inner mitochondrial membrane maintains the proton gradient across the membrane, and is another source of ATP production through oxidative phosphorylation (Nolfi-Donegan et al., 2020). The ETC is comprised of complexes I-V, all of which have substrates for SIRT3. SIRT3-mediated deacetylation increases the efficiency of the ETC (Novgorodov et al., 2016; Gleave et al., 2017), which is particularly important in neurons, as they require more energy compared to other cell types as evidenced by the brain accounting for over 20% of oxygen consumption, of which 75–80% is allocated to neurons (Hyder et al., 2013). This energetic demand, combined with neurons being limited to aerobic metabolism unlike other high energy demanding cells like muscles, makes neurons more sensitive to changes in homeostasis, as insufficient energy supply can impair normal neuronal function (Vergara et al., 2019). In non-neuronal cells, SIRT3 appears to increase ATP, whereas in certain populations of neuronal cells, SIRT3 reduces the metabolic demand of neurons, thus decreasing the requirement for ATP (Hirschey et al., 2010; Gleave et al., 2017; Zhang et al., 2017). Within mitochondria complex I, SIRT3 deacetylates two subunits, NDUFA11 and NDUFS8, leading to the dehydrogenization of NADH to NAD+ (Lombard et al., 2007). Interestingly, the production of NAD+ by complex I may increase the activity of SIRT3 as its deacetylation activity is NAD + -dependent (Yang et al., 2007, 2010; Magnifico et al., 2013). Complex II of the ETC transfers electrons from succinate to ubiquinone, and also plays a role in the TCA cycle by catalyzing the oxidation of succinate to fumarate (Finley et al., 2011; Kristian et al., 2021). SIRT3 deacetylates SDHA and SDHB, two of four subunits of complex II, which increases complex II activity (Cimen et al., 2010; Finley et al., 2011). However, it appears that complex II mediated ATP production can proceed even in the absence of SIRT3, suggesting SIRT3 is involved in the regulation of complex II activity but is not the sole regulatory protein (Ahn et al., 2008; Parodi-Rullan et al., 2018). In contrast, deacetylation of complex III by SIRT3 results in an increase in ATP production in the ETC, with the loss of SIRT3 having a negative impact on complex III activity and therefore ATP production (Kim et al., 2010). Complex III is also an important contributor to cellular ROS in the ETC, making its regulation by SIRT3 crucial in maintaining both ATP homeostasis as well as oxidative stress regulation (Bell et al., 2011). Furthermore, a number of acetylation sites have been identified in complex IV that are targets of SIRT3, with their deacetylation attenuating apoptosis as a result of oxidative stress in neuronal cells (Tu et al., 2019). Specifically, inhibition of SIRT3 by siRNA resulted in the acetylation of cytochrome c oxidase-1 (COX-1), which is a subunit of mitochondrial complex IV, leading to a loss of mitochondrial membrane potential (Tu et al., 2019). The interaction between SIRT3 and complex IV is not as well studied, and further studies are required to determine the effects of complex IV deacetylation by SIRT3 on mitochondrial stability. Lastly, complex V deacetylation by SIRT3 has been shown to regulate ATP production in human 143B cells and cardiac cells, where knockdown of SIRT3 results in elevated acetylation levels of the α and OSCP subunits of complex V (Wu et al., 2013). Furthermore, honokiol-induced increases in SIRT3 expression in mice augmented ATP production through the deacetylation of complex V, respectively, (Rahman et al., 2014; Vassilopoulos et al., 2014; Zhang et al., 2016). Taken together, these studies show that, SIRT3 plays a key role in the regulation of oxidative phosphorylation through reversible deacetylation.

### SIRT3 regulates oxidative stress

Elevated ROS levels induce biomolecular damage, such as lipid peroxidation of the cellular membrane, protein oxidation, DNA damage, neuroinflammation and excitotoxicity, and cumulative damage of mitochondria, which disrupts cellular processes, and plays a large role in cellular aging. This may ultimately lead to degeneration of neuronal cells (Chen et al., 2012; Stefanatos and Sanz, 2018). In dopaminergic neurons, SIRT3 expression is elevated in response to increases in oxidative stress to promote mitochondrial function (Lee et al., 2021) through regulating antioxidant processes as later described. The antioxidant activity promoted by SIRT3 plays a crucial role in mitigating the detrimental effects of oxidative stress, serving as important cytoprotective and longevity enhancing functions (Singh et al., 2018). As the major deacetylase of the mitochondria, endogenous SIRT3 is known to directly activate protein substrates involved in the production and detoxification of ROS (Cheng et al., 2016; Zhang X. et al., 2016; Figure 2). Important substrates for SIRT3 which regulate oxidative stress in the mitochondria include MnSOD, GDH, FOXO3a, SDH, and IDH2, as well as other substrates on mitochondria complexes (Kops et al., 2002; Sundaresan et al., 2009; Cimen et al., 2010; Hafner et al., 2010; Tao et al., 2010; Finley et al., 2011; Yu et al., 2012; Li et al., 2015; Yang Y. et al., 2016; Jiang et al., 2017; Fu et al., 2022). In SIRT3 KO mouse models, hyperacetylation of mitochondrial proteins including manganese superoxide dismutase (MnSOD) and the metabolic enzyme glutamate dehydrogenase (GDH) is evident, compared to WT (Lombard et al., 2007; Tao et al., 2010). MnSOD allows for the clearance of ROS as it catalyzes the reduction of ROS into hydrogen peroxide that is later converted into water and oxygen by the protein catalase (Tao et al., 2010; Trinh et al., 2021). This finding is consistent with the *in vitro* studies in SIRT3 KO mouse embryonic fibroblasts (MEFs) infected with lentivirus vectors expressing oncogenes Myc or Ras, which have elevated levels of oxidative stress that could be attenuated by adenoviral overexpression of MnSOD (Kim et al., 2010). Furthermore, in dopaminergic primary cultured neurons, SIRT3 reduces oxidative stress by increasing the activity of MnSOD through deacetylation at its K122 or K130 sites (Tao et al., 2010; Zhang et al., 2016). Additionally, SIRT3 deacetylates and activates IDH2 in HEK293 cells (Lombard et al., 2007; Yu et al., 2012). The deacetylation of IDH2 catalyzes the oxidative decarboxylation of isocitrate to 2-oxoglutarate, an intermediate substrate of the TCA cycle, by utilizing nicotinamide adenine dinucleotide phosphate (NADP+) as a cofactor (Schlicker et al., 2008; Someya et al., 2010). Downregulation or IDH2 insufficiency in mice leads to the accumulation of mitochondrial ROS, and has been associated with impaired mitophagy and wound healing (Kim and Park, 2019).

SIRT3 also deacetylates glutamate dehydrogenase (GDH), increasing the conversion of glutamate to α-ketoglutarate and ammonia, while reducing NADP+ to NADPH in the process (Francis et al., 2014). The increased levels of NADPH allow for the enzyme glutathione reductase to convert oxidized glutathione to reduced glutathione (GSH) (Lopert and Patel, 2014). This in turn is used as a cofactor by glutathione peroxidase for the clearance of ROS (Lombard et al., 2007; Schlicker et al., 2008; Lopert and Patel, 2014).

SIRT3 also targets FOXO3a, increasing the expression of proteins involved in cell survival and stress resistance (Jacobs et al., 2008; Sundaresan et al., 2009; Peserico et al., 2013; Tseng et al., 2013). As evidenced in BV-2 cell lines, overexpression of exogenous SIRT3 leads to the increase in protein levels and nuclear localization of FOXO3a corresponding to the increase in the expression of antioxidants such as catalase and MnSOD (Rangarajan et al., 2015). This has been shown to reduce cellular levels of ROS, offering overall protection against stressors such as cardiac hypertrophy, where protection is lost in SIRT3 KO mice as the absence of SIRT3 leads to an increase in mitochondrial ROS accumulation during cardiac hypertrophy (Sundaresan et al., 2009). Furthermore, the deacetylation of FOXO3a upregulates the expression of PGC-1α, a positive regulator of SIRT3 expression (Kong et al., 2010; Tseng et al., 2013). Therefore, SIRT3 can upregulate its own expression in response to cellular stress in order to protect the cell from further damage induced by oxidative stress.

### SIRT3 regulates mitochondrial dynamics

**Figure 3:**
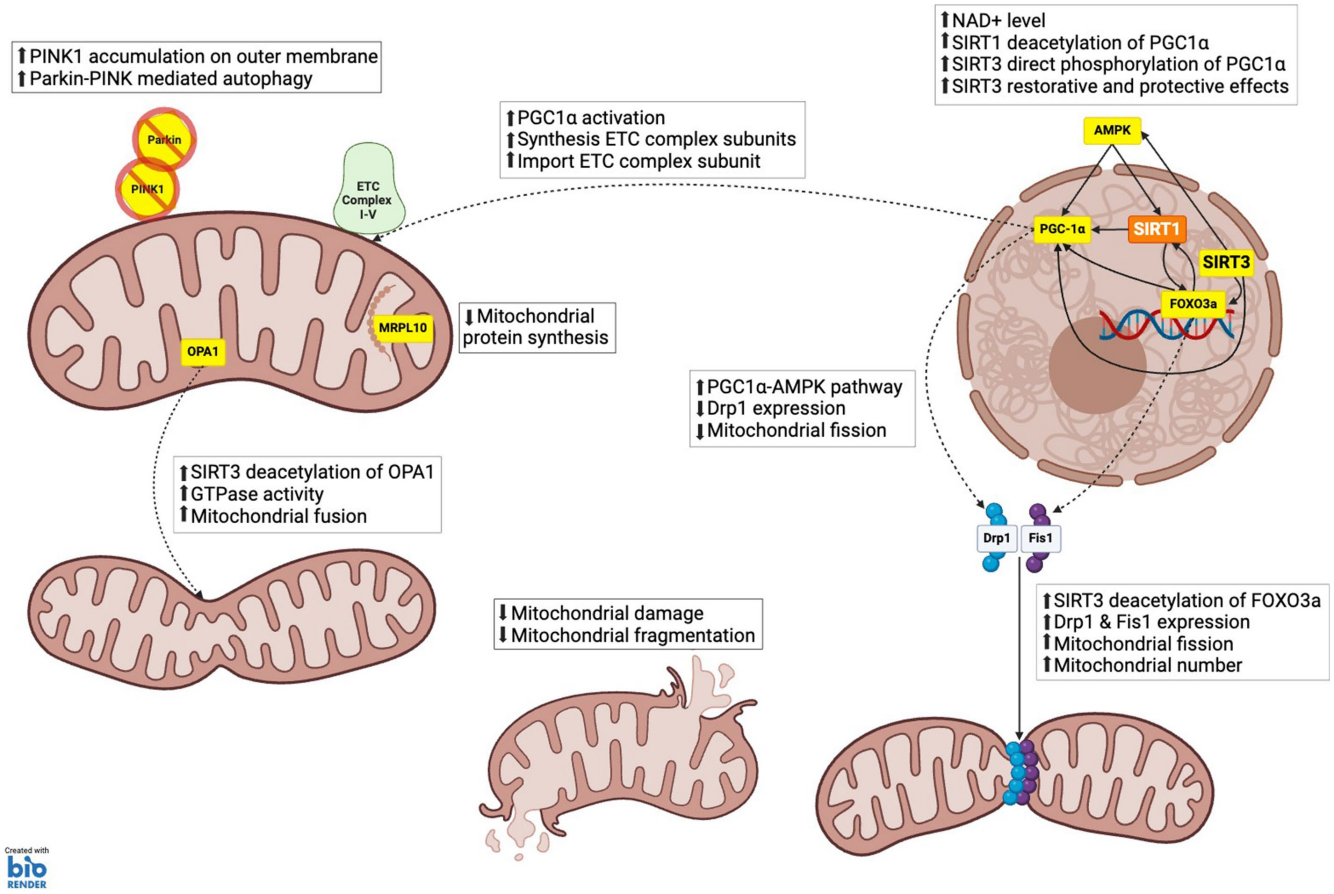
SIRT3 role in mitochondrial dynamics. **(1) MITOCHONDRIA BIOGENESIS:** PGC-1α, which enhances the synthesis of electron transport chain (ETC) complex subunits and their import into mitochondria, facilitating increased mitochondrial capacity and function; AMPK via acetyl-CoA synthetase 2 (ACS2) activation, which phosphorylates and activates PGC-1α, thereby promoting mitochondrial biogenesis. **(2) MITOCHONDRIA FISSION AND FRAGMENTATION:** FOXO3a, which upregulates the expression of dynamic-related protein 1 (Drp1) and mitochondrial fission 1 protein (Fis1). These proteins are essential for the fission process, enabling the division of mitochondria and thereby increasing mitochondrial number. Mitochondrial fusion: Optic atrophy 1 (OPA1), which increases GTPase activity, promoting the fusion of mitochondrial membranes, and maintaining mitochondrial crista structure. **(3) MITOCHONDRIA PROTEIN SYNTHESIS:** Mitochondrial ribosomal protein L10 (MRPL10), reducing the production of mitochondrial proteins. This regulation conserves energy under stress conditions by decreasing the synthesis of electron transport chain components. **(4) MITOPHAGY AND AUTOPHAGY:** SIRT3 supports mitophagy by enhancing the stability and activity of proteins like PTEN-induced kinase 1 (PINK1) and Parkin through deacetylation. This ensures efficient clearance of damaged mitochondria and prevents the accumulation of dysfunctional mitochondrial components.

Mitochondrial dynamics refers to the processes by which healthy levels of mitochondria are maintained, and involve mitochondria biogenesis, fission, and fusion, as well as mitochondrial breakdown through mitophagy (Figure 3). Under basal conditions, SIRT3 can bind to ATP synthase to promote ATP production through the ETC (Yang W. et al., 2016). However, with the loss of mitochondrial membrane potential and subsequent drop in pH which can be caused by mitochondrial damage, SIRT3 dissociates from ATP synthase to promote mitochondrial health (Yang W. et al., 2016). SIRT3 promotes mitochondrial biogenesis through direct deacetylation of optic atrophy 1 (OPA1), which mediates mitochondrial fusion as well as maintaining crista structure and protecting cells from apoptosis (Samant et al., 2014; Benigni et al., 2019). In SIRT3 KO murine embryonic fibroblast (MEFs), hyperacetylation of OPA1 is observed and this was shown to reduce the GTPase activity of the protein compared to WT controls (Samant et al., 2014). SIRT3 overexpression in WT MEFs resulted in a filamentous mitochondrial network whereas OPA1 KO MEFs overexpressing mito-GFP or SIRT3 using adenovirus did not exhibit this effect (Samant et al., 2014). Therefore, SIRT3 is thought to modulate mitochondrial morphology, which is dependent on the presence of Optic Atrophy 1 (OPA1) (Samant et al., 2014; Signorile et al., 2017; Benigni et al., 2019). In COS7 cells, using quantitative deacetylation assays with recombinant, purified SIRT3, as well as Western blotting and mass spectrometry, it was shown that SIRT3 activates AMPK via activation of AceCS2 (Hallows et al., 2006). This allows for the direct phosphorylation of PGC-1⍺ or the deacetylation of PGC-1α by SIRT1 promoted by the increased NAD+ levels (Zhou et al., 2017). The AMPK, PGC-1α-SIRT1-SIRT3 cycle has been linked with many restorative and protective effects (Wareski et al., 2009; Peserico et al., 2013; Zhang X. et al., 2016; Dai et al., 2017; Zhou et al., 2017). For example, activated PGC-1α interacts with nuclear respiratory factor 1/2 (NRF1/2) to activate mitochondrial transcription factor A (TFAM) and promote the synthesis and import of nuclear-encoded ETC complex subunits to the mitochondria (Scarpulla, 2002; Hallows et al., 2006; Uittenbogaard and Chiaramello, 2014).

SIRT3 also affects mitochondrial morphology through the regulation of mitochondrial fission (Song et al., 2013; Tseng et al., 2013). In human umbilical vein endothelial cells (HUVECs) transfected with SIRT3 short interfering RNA (siRNA), reduced levels of dynamin-related protein 1 (Drp1), mitochondrial fission 1 protein (Fis1) and mitofusin 2 (Mfn2) were detected relative to the deacetylation deficient SIRT3 control (SIRT3 H248Y) (Tseng et al., 2013). This is the indirect result of SIRT3 deacetylating FOXO3a, which upregulates Drp1 and Fis1 expression (Tseng et al., 2013). Drp1 and Fis1 facilitate the process of mitochondrial fission, and therefore their downregulation would result in a reduction in mitochondrial fission and overall mitochondrial number. Conversely, in mouse hepatocytes, SIRT3 overexpression appears to downregulate Drp1 expression through the AMPK-PGC-1α pathway, since SIRT3 shRNA increases Drp1 expression, suggesting that SIRT3 can both positively and negatively regulate fission through Drp1 (Liu et al., 2017). It has also been proposed that SIRT3 mediates these effects through the liver kinase B1 (LKB1)-AMPK pathway. In HK-2 cells, SIRT3 siRNA prevents SIRT3-mediated deacetylation of LKB1, which would normally promote the phosphorylation of AMPK by LKB1 and downstream phosphorylation of Drp1 (Mao et al., 2022). Therefore, it appears that SIRT3 can regulate mitochondrial fission through independent pathways that can either promote or inhibit fission.

SIRT3 also plays a role in mitochondrial fusion (Samant et al., 2014; Jian et al., 2023). The mitochondrial fusion protein, OPA1 is deacetylated by SIRT3 resulting in an increase of its GTPase activity, which increases the rate of mitochondrial fusion (Samant et al., 2014; Jian et al., 2023). OPA1 is anchored to the inner membrane of the mitochondria, where it becomes hyperacetylated under conditions of cellular stress. In a mouse model of diabetes, this hyperacetylation is reduced through SIRT3 deacetylation to restore GTPase activity as evidenced *in vivo* (Samant et al., 2014). In addition, SIRT3 targets and deacetylates i-AAA protease (YME1L1), an upstream regulator of OPA1 (Jian et al., 2023). In renal tubular epithelial cells (TEC), overexpression of SIRT3 attenuated mitochondrial damage caused by lipopolysaccharide (LPS)-induced stress through the promotion of OPA1-mediated mitochondrial fusion (Jian et al., 2023).

SIRT3 also regulates mitophagy through the regulation of several pathways (Tseng et al., 2013; Qiao et al., 2016; Yu et al., 2017). NIP-like protein X (NIX), FUN14 domain containing 1 (FUNDC1), FK506-binding protein 8 (FKBP8), and BCL2-like 13 (BCL2-L13) are mitophagy receptors, sometimes referred to as BCL2 interacting protien 3 (BNIP3) and BCL2 interacting protein 3 like (BNIP3L), which target mitochondria to autophagosomes through the direct interaction with autophagy-related protein 8 (ATG8) proteins (O'Sullivan et al., 2015; Wang et al., 2018; Onishi et al., 2021). SIRT3 increases the levels of BNIP3 and BNIP3L downstream through the deacetylation of FOXO3a, promoting mitophagy to improve mitochondrial health in response to oxidative stress (Wu et al., 2020). In HUVECS, knockdown of SIRT3 results in decreased levels of BNIP3, NIX and LC3-II/LC3-I, which is reversed by the overexpression of FOXO3a (Tseng et al., 2013). Alternatively, BNIP3 can be upregulated by SIRT3 via the activation of the extracellular signal-regulated kinase (ERK)-cAMP response element-binding protein (CREB) pathway to maintain mitophagy, as shown in hepatocytes where blocking the ERK-CREB pathway negated the positive effects of SIRT3 overexpression on BNIP-mediated mitophagy (Li et al., 2018). Mitophagy can also occur through the PTEN induced kinase 1 (PINK1)/Parkin-dependent pathway, where a decrease in mitochondrial membrane potential leads to the accumulation of PINK1 on the outer mitochondrial membrane, and phosphorylation of Parkin (Geisler et al., 2010; Matsuda et al., 2010; Greene et al., 2012; Kazlauskaite et al., 2014; Jacoupy et al., 2019). This leads to the ubiquitination of proteins found on the outer mitochondrial membrane to promote the interaction with autophagosomes (Oshima et al., 2021). Deacetylation of PINK1 or Parkin by SIRT3 prevents the breakdown of PINK1 and promotes Parkin phosphorylation, thus promoting mitophagy (Auburger et al., 2014; Wei et al., 2017). SIRT3 can also indirectly induce Parkin-mediated mitophagy through the deacetylation of FOXO3a and AMPK activation, which increases Parkin expression and phosphorylation, respectively, (Wei et al., 2017; Yu et al., 2017).

### The role of SIRT3 in regulation of proteostasis

SIRT3 plays a critical role in maintaining proteostasis in the cell through the regulation of protein synthesis and degradation (Figure 3). This process is partly under the control of heat shock protein 60 (HSP60), which is a major mitochondrial chaperone that is involved in maintaining mitochondrial proteostasis (Wang X. et al., 2016). SIRT3 levels are lowered through protein degradation in the absence of HSP60, indicating that HSP60 is required for promoting the stability of SIRT3 (Wang X. et al., 2016). Omentin1, an adipokine that allows crosstalk between adipose tissue and other organs, increases mitochondrial fusion while decreasing fission through activation of SIRT3 (Hu et al., 2022). Within the mitochondria, SIRT3 reversibly deacetylates ribosomal protein mitochondrial ribosomal protein L10 (MRPL10), specifically at the N-terminal domain of the protein, inhibiting the synthesis of mitochondrial proteins (Yang et al., 2010). This was shown in SIRT3 KO mice, where in isolated mitochondrial ribosomes, MRPL10 was more acetylated in comparison to WT mice (Yang et al., 2010). Mitochondrial protein synthesis is required for the synthesis of 13 proteins, specifically subunits of the ETC, which include 6 subunits of complex I (ND1-6), cytochrome *b*, 3 subunits of complex IV (COI-III), and 2 subunits of complex V (ATP6 and ATP8) (Yokokawa et al., 2018; Wang et al., 2021). In C2C12 myoblast cells with shRNA-induced SIRT3 knockdown, elevated levels of mitochondrial protein synthesis were observed (Yang et al., 2010). During periods of cellular stress, downregulation of protein synthesis may prove beneficial to cells, as it may allow for the allocation of energy to reduce oxidative stress.

SIRT3 also regulates proteostasis through modulation of protein degradation (Papa and Germain, 2014; Samant et al., 2014). Specifically, this occurs through the mitochondrial unfolded protein response (mtUPR), which modifies protein folding to prevent the accumulation of abnormal proteins in the mitochondria (Papa and Germain, 2014). The phosphorylation of AMPK, possibly through the deacetylation of LKB1 mediated by SIRT3 expression, has been found to upregulate the expression of mtUPR-related genes encoding proteins such as caseinolytic mitochondrial matrix peptide proteolytic subunit (CLPP), HSP60, and lon peptidase 1 (LONP1) (Xu M. et al., 2019; Chen et al., 2021). CLPP is a serine protease that contributes to the degradation of proteins and inhibits the phosphorylation of α-synuclein, which normally promotes the misfolding of α-synuclein (Hu et al., 2019). LONP1 is a serine protease responsible for the selective degradation of misfolded, incomplete, or oxidatively damaged proteins in the mitochondrial matrix (Chen et al., 2021). On the other hand, HSP60 acts as a chaperone protein required for the folding of polypeptides into proteins and complexes, as well as preventing the aggregation of proteins following heat shock (Chen et al., 2021). Lastly, the mtUPR can also be activated through the translocation of sphingosine kinase 1 (Sphk1) to the outer mitochondrial membrane, as the phosphorylation of sphingosine into sphingosine-1-phosphate promotes mtUPR activity (Xiaowei et al., 2023). Sphk1 protein stability is regulated by FOXO3. Importantly, SIRT3-mediated deacetylation prevents degradation of FOXO3, thus maintaining the mtUPR by stabilizing Sphk1 (Tseng et al., 2014; Xiaowei et al., 2023). Conversely, deacetylation of proteins by SIRT3 can also promote degradation, such as in the case of the tumor suppressor protein p53, where deacetylation results in the degradation of the protein through the ubiquitin proteasome system (UPS) (Xiong et al., 2018).

### SIRT3-mediated regulation of immune responses

SIRT3 has also been implicated in immunological responses throughout the body (Warren and MacIver, 2019; Fortuny and Sebastian, 2021). In response to limited glucose, AMPK activation leads to the activation of SIRT3 through sentrin-specific protease 1 (SENP1) to promote T cell survival and memory development (He et al., 2021). Conversely, inflammatory stress can activate SIRT3 which reduces the effects of inflammation on microglia, inhibited by the presence of miRNA-494 in the brain (He et al., 2022). Whether these effects are directly linked with the ability of SIRT3 to regulate mitochondrial function is unknown. In SIRT3 KO T cells isolated from mice spleen, in a mixed lymphocyte reaction (MLR), reduced proliferation and cytokine production of effector T cells was observed compared to WT T cells in response to both nonspecific T-cell receptor (TCR) and allogeneic stimulation (Toubai et al., 2018). Interestingly, CD4+/CD8+ SIRT3 KO T cells exhibit reduced production of ROS compared to WT T cells after both nonspecific TCR and allostimulation, with no difference in mitochondrial content between SIRT3 KO and WT T cells (Toubai et al., 2018). These findings are contrary to the expected role of SIRT3 in inhibiting ROS production as discussed earlier, and thus suggest a unique function of SIRT3 in modulating ROS production in T cells during immune responses (Toubai et al., 2018). Furthermore, in regulatory T cells, SIRT3 KO impaired allograft survival due to the inhibition of oxidative phosphorylation compared to WT regulatory T cells (Beier et al., 2015). SIRT3 also acts as a tumor suppressor in B cells, specifically malignant B cells from patients where SIRT3 expression is reduced compared to healthy patients (Yu et al., 2016). Thus, in malignant B cell lines, overexpression of SIRT3 results in the deacetylation of IDH2 and MnSOD, which are hyperacetylated in malignant B cells (Yu et al., 2016). In addition, SIRT3 promotes the polarization of macrophages through the deacetylation of FOXO1, which prevents the apoptosis of HK-2 cells due to renal calcium oxalate crystal formation (Xi et al., 2019).

## The role of SIRT3 in regulation of the blood-brain barrier

The blood–brain barrier (BBB) consists of endothelial cell, pericytes, capillary basement membrane, and astrocyte end-feet, all of which serve to filter toxins and harmful compounds from the brain within the bloodstream, and supply brain tissue with oxygen and nutrients. The BBB deteriorates with increasing age, which is believed to contribute to cognitive impairment, neurodegeneration and disease. While it is largely unknown how the BBB deteriorates, loss of SIRT3 function appears to be a contributing factor. Firstly, in the BBB, SIRT3 declines with age and in AD (Cheng et al., 2020; Tyagi et al., 2021). Also, in an ischemic stroke model, SIRT3 KO mice showed more severe BBB leakage and inflammatory responses compared with WT mice. Furthermore, using GFAP as a promoter, virally-mediated SIRT3-selective transduction in astrocytes showed BBB protection (Yang et al., 2021). These effects are thought to be caused through SIRT3-mediated inhibition of hypoxia inducible factor-1α (HIF-1α), which regulates vascular endothelial growth factor (VEGF) expression after ischemia. Furthermore in the BBB, SIRT3 increases production of the tight junction proteins ZO1 and Claudin 5, which has also been shown to promote BBB integrity. In ischemic mouse models, SIRT3 activation through monocyline and honokiol increases VEGF, ZO1 and Claudin 5 levels, where as SIRT3 knockdown using siRNA reduces ZO1 and Claudin 5 (Tyagi et al., 2021; Yang et al., 2021).

Recently, using single-nucleus transcriptomics in mice, decreased connexin 43 (CX43) expression in cadherin-5^+^ (Cdh5^+^) cerebral vascular cells was identified as a factor contributing to age-related decline of the BBB. This was confirmed in human brain samples. The CX43-dependent effect was caused by reduced NAD^+^ levels and loss of SIRT3 causing mitochondrial dysfunction. This study showed that CX43 negatively regulates poly(ADP-ribose) polymerase 1 (PARP1), and that blockade of PARP1, or nicotinamide mononucleotide (NMN) supplementation rescues NAD and alleviates BBB leakage that occurs with increasing age (Zhan et al., 2023).

## Summary and conclusion

SIRT3 is a nutrient biosensor and mitochondrial protein deacetylase with many mitochondrial substrates. Through de-acetylation of these substrates, SIRT3 regulates many aspects of mitochondrial function, including playing a crucial role in mitochondrial metabolism, where it helps to control the TCA cycle and fatty acid oxidation, regulating energy production. SIRT3 also stabilizes the ETC, further controlling energy production, as well as reducing free radical production. Furthermore, SIRT3 controls mitochondrial dynamics through regulation of biogenesis, fission, fusion and mitophagy. These important and wide-spanning mitochondrial functions result in SIRT3 having the ability to dramatically influence cellular homeostasis, including proteostasis and immune function. Because of the important role of SIRT3 in regulating mitochondrial function and homeostasis, it has been shown to play crucial roles in energy demanding organs and tissues, such as the heart, liver, brain and skeletal muscle. Indeed, there is a large body of evidence to suggest that increasing SIRT3 activity has protective effects, which has been shown to be of therapeutic benefit in cell and animal models of cardiovascular, renal, liver and neurodegenerative diseases (Gleave et al., 2017; Morigi et al., 2018; He et al., 2019; Govindarajulu et al., 2021; Trinh et al., 2023). Emerging studies are now focusing on non-invasive and selective ways of increasing SIRT3, such as focused ultrasound-induced opening of the BBB to deliver SIRT3 as a gene therapy (Trinh et al., 2022).

## Author contributions

DT: Writing – original draft, Writing – review & editing, Conceptualization. LA: Writing – review & editing, Conceptualization. HB: Writing – original draft. MK: Writing – original draft. JN: Conceptualization, Writing – original draft, Writing – review & editing.

## Glossary

AceCS2: Acetyl-CoA synthetase 2
AMPK: Adenosine monophosphate-activated protein kinase
ATG8: Autophagy-related protein 8
ATP: Adenosine triphosphate
BCL2-L13: BCL2-like 13
BMDCs: Bone marrow-derived dendritic cells
BNIP3: BCL2 interacting protein 3
cAMP: Cyclic adenosine monophosphate
CNS: Central nervous system
CoQ10: Co-enzyme Q10
Drp1: Dynamin related protein1
ETC: Electron transport chain
Fis1: Mitochondrial fission 1 protein
FOXO3a: Forkhead box O3a
FUNDC1: FUN14 domain containing 1
GDH: Glutamate dehydrogenase
GST: Glutathione S-transferase
HIF-1: Hypoxia inducible factor 1
HSP: Heat shock protein
HUVECs: Human umbilical vein endothelial cells
Iba1: Ionized calcium binding adaptor molecule 1
IDH2: Isocitrate dehydrogenase 2
kB: Nuclear factor kappa B
IkBα: NF-kB
IOP: Intraocular pressure
LKB1: Liver kinase B1
LONP1: Lon peptidase 1
MEFs: Murine embryonic fibroblast
Mfn2: Mitofusin 2
MLR: Mixed lymphocyte reaction
MnSOD: Manganese superoxide dismutase
MRPL10: Mitochondrial ribosomal protein L10
mtUPR: Mitochondrial unfolded protein response
NAD: Nicotinamine adenine dinucleotide
NADPH: Nicotinamide adenine dinucleotide phosphate
NIX: NIP-like protein X
NMN: Nicotinamide mononucleotide
NR: Nicotinamide riboside
NRF1/2: Nuclear respiratory factor 1/2
ONL: Outer nuclear layer
OPA1: Optic atrophy 1
OTC: Ornithine transcarbamylase
PGC-1α: Peroxisome proliferator-activated receptor gamma coactivator 1-alpha
ROS: Reactive oxygen species
KO: Knockout
SDH: Succinate dehydrogenase
SDHA: Succinate dehydrogenase flavoprotein
SDHB: Succinate dehydrogenase iron-sulfur protein
shRNA: Short hairpin RNA
siRNA: Short interfering RNA
SIRT: Sirtuin
SNP: Single nucleotide polymorphism
SOD2: Superoxide dismutase 2
TCA: Tricarboxylic acid cycle
TCR: T-cell receptor
Teff: Effector T cell
TFAM: Mitochondrial transcription factor A
Treg: Regulatory T cells
UCP2: Mitochondrial uncoupling protein 2
VNTR: Variable number of tandem repeats
WT: Wild-type
